# International Leprosy Conference, Berlin, 1897

**Published:** 1898-04

**Authors:** 


### INTERNATIONAL LEPROSY CONFERENCE, BERLIN, 1897. GENERAL CONCLUSIONS.

At the close of the debates of the International Leprosy Conference, Berlin, 1897, the secretaries have the honor to present the following short report of the general conclusions of the conference:

They believe that such a resume will be especially desirable for those members who have been delegated by their respective governments, and who have to make reports on the results of the conference.

As might be expected, a considerable portion of the discussion has related to the bacillus lepræ, which the conference accepts as the virus of leprosy, and which for upwards of twenty-five years has been known to the scientific world through the important discovery of Hansen and the able investigations of Neisser.

The conditions under which the bacillus grows and develops are still unknown, as well as the way of its invasion into the human system; but from the discussions of the conference, it seems probable that an unanimity of opinion will soon prevail in reference to its modes of subsequent dissemination within the human body.

Very interesting observations have been brought forward in connection with the elimination of the bacilli in large quantities by means of the skin and the nasal and buccal mucous membranes of lepers; it is desired that such observations be confirmed where opportunities occur.

The question is of very great importance to those who are entrusted with the care of the public health, as leprosy is now acknowledged to be a contagious disease.

Every leper is a danger to his surroundings, the danger varying with the nature and extent of his relations therewith, and also with the sanitary conditions under which he lives.

Although among the lower classes, every leper is especially dangerous to his family and fellow-workers, cases of leprosy frequently appear in the higher social circles.

The theory of heredity of leprosy is now further shown to have lost ground, in comparison with the at present generally accepted theory of its contagiousness.

The treatment of leprosy has only had palliative results up to the present time.

Serum therapy has so far been unsuccessful.

In view of the virtual incurability of leprosy and the serious and detrimental effects which its existence in a community causes, and considering the good results which have followed the adoption of legal measures of isolation in Norway, the leprosy conference, as a logical issue of the theory that the disease is contagious, has adopted the following resolution proposed by Dr. Hansen and amended by Dr. Besnier.

1. In such countries, where leprosy forms foci or has a great extension, we have in isolation the best means of preventing the spread of the disease.

2. The system of obligatory notification, of observation and isolation as carried out in Norway, is recommended to all nations with local self-government and a sufficient number of physicians and health officers.

3. It should be left to the legal authorities after consultation with the medical authorities, to take such measures as are applicable to the special social conditions of the districts.

Secretaries of the conference:

Phin. S. Abraham, London; E. Dubois Havenith, Bruxelles; Ed. Arning, Hamburg; J. J. Kinyoun, Washington; A. von Bergmann, Riga; G. Thibierge, Paris; Edv. Ehlers, Copenhagen, General Secretary.

---

Messrs. LEA BROTHERS & CO. announce for early publication the following books by eminent authorities. Complete catalogues of the publications of this firm may be had by addressing either their Philadelphia or New York house.

A Manual of Otology.—By Gorham Bacon, A.M., M.D., Professor of Otology in University Medical College, New York. With an Introductory Chapter by Clarence J. Blake, M. D., Professor of Otology in the Harvard Medical School, Boston, Mass. In one handsome 12mo, volume, with numerous illustrations.

The Treatment of Surgical Patients Before and After Operation.—By Samuel M. Brickner, M. D., Visiting Surgeon at the Mt. Sinai Hospital, New York. In one handsome volume of about 400 pages, with illustrations.

A Text-Book of Dental Pathology, Therapeutics, and Pharmacology.—Being a Treatise on the Principles and Practice of Dental Medicine. By Henry H. Burchard, M.D., D.D.S., Special Lecturer on Dental Pathology and Therapeutics at the Philadelphia Dental College, Philadelphia. In one handsome octavo volume of about 550 pages, with 400 illustrations.

The Principles of Treatment.—By J. Mitchell Bruce, M. D., F. R. C. P., Physician and Lecturer on Materia Medica and Therapeutics at Charing-Cross Hospital, London. In one octavo volume.

Diseases of the Nose, Throat, Naso-Pharynx, and Trachea.—A Manual for Students and Practitioners. By Cornelius G. Coakley, M. D., Professor of Laryngology in University Medical College, New York. In one volume, 12mo, of about 400 pages, with numerous illustrations, many of which are in colors.

Diseases of Women.—A Manual of Non-surgical Gynecology, designed especially for the use of Students and General Practitioners. By Francis H. Davenport, M. D., Instructor in Gynecology in the Medical Department of Harvard University, Boston. Third edition, thoroughly revised and enlarged, with many additional illustrations.

A Treatise on Gynecology.—By E. C. Dudley, A. M., M. D., Professor of Gynecology in the Chicago Medical College, Chicago. In one octavo volume of about 600 pages, with 425 illustrations, many of which are in colors.

A Text-Book of Anatomy—By American Authors. Edited by Frederic Henry Gerrish, M. D., Professor of Anatomy in the Medical School of Maine. In one handsome imperial octavo volume, copiously illustrated in colors.

Manual of Skin Diseases.—With Special Reference to Diagnosis and Treatment. For the Use of Students and General Practitioners.By W. A. Hardaway, M. D., Professor of Skin Diseases inthe Missouri Medical College. Second edition, entirely rewritten and much enlarged. In one handsome 12mo, volume with illustrations.

The Principles and Practice of Obstetrics.—By American Authors. Edited by Charles Jewett, M. D., Professor of Obstetrics in the Long Island College Hospital, Brooklyn, N. Y. In one handsome octavo volume, with many illustrations in black and in colors.

LEA BROTHERS & CO., PUBLISHERS,
706, 708, 710 Sansom Street, Philadelphia.
111 Fifth Avenue (cor 18th St.), New York.

---

MALARIA HOLLOW, April 1st, 1898.

DEAR DOKTOR: i am mighty obliged to you fer the loan uv thet stone fer it wus luky thet i hed it fer wen i operated on old man Kalkuli i found he dident hev no stones thet is he hed none in his blader. say doc i hed an operation this mornin thet is to say i operated on young Mrs. Eggly she had a pain in hur overy an i bin trin to git hur to let me kut thet overy out. wal it hurted hur so much thet at last she sed go a hed and kut it out. wal you see it bein the furst time i kut overies out uv a woman i got kinder mixed and after she wus under kloroform i furgot which overy it wus thet hurted hur but i went a hed an kut both uv them out fur i knowed she wud never no the diffurence an if she komplaned uv both sides hurtin after the operation i kud tell hur the other side hurt from simpithy. say doc kuttin overies out is ded easey. the trouble is tu git the pachunt over the afekts uv the operachun. say doc im writin an artikle on intubation uv the rektum fur kronic flatulency to be published in the beanville medical Bazoo. Dr. Cuttinskope es bin sick he wus pasin gaul stones. i told every bodie he hed hog kolera an yu ken bet he wus mad. wal i dont think Cuttinskope will stay hear long any more kos the folks round hear dont count much on his durn spiglass biznis. they want a doctor what knows what to do with out runnin home to look through a mikroskope just like ole man Hayrick sed he dident give a durn fur siense what he wanted wus relief. whats the gud uv makin them durn bugs so big you ken see em with a spiglass you kant hit em with an ax and kill em any way. i guess ile jes keep on killin them bugs with pisen with out seain them. wal doc so long until i rite a gain. Your esteemin friend, Dr. M. T. Head, M. D.

